# Supplementary material for: The oncogenic fusion protein DNAJB1-PRKACA can be specifically targeted by peptide-based immunotherapy in fibrolamellar hepatocellular carcinoma
Source: Nat Commun. 2022 Oct 27;13:6401. doi: 10.1038/s41467-022-33746-3 (PMC9613889; doi:10.1038/s41467-022-33746-3)
Supplement: Supplementary file 1 — Supplementary Information [file 41467_2022_33746_MOESM1_ESM.pdf]

Supplementary information for

The oncogenic fusion protein DNAJB1-PRKACA can be actively targeted by peptide-based immunotherapy in fibrolamellar hepatocellular carcinoma

Jens Bauer, Natalie Köhler, Yacine Maringer, Philip Bucher, Tatjana Bilich, Melissa Zwick, Severin Dicks, Annika Nelde, Marissa Dubbelaar, Jonas Scheid, Marcel Wacker, Jonas S. Heitmann, Sarah Schroeder, Jonas Rieth, Monika Denk, Marion Richter, Reinhild Klein, Irina Bonzheim, Julia Luibrand, Ursula Holzer, Martin Ebinger, Ines B. Brecht, Michael Bitzer, Melanie Boerries, Judith Feucht, Helmut R. Salih, Hans-Georg Rammensee, Stephan Hailfinger, Juliane S. Walz

This Supplementary information includes:

Supplementary Figures S1 to S10

Supplementary Tables 1 to 5

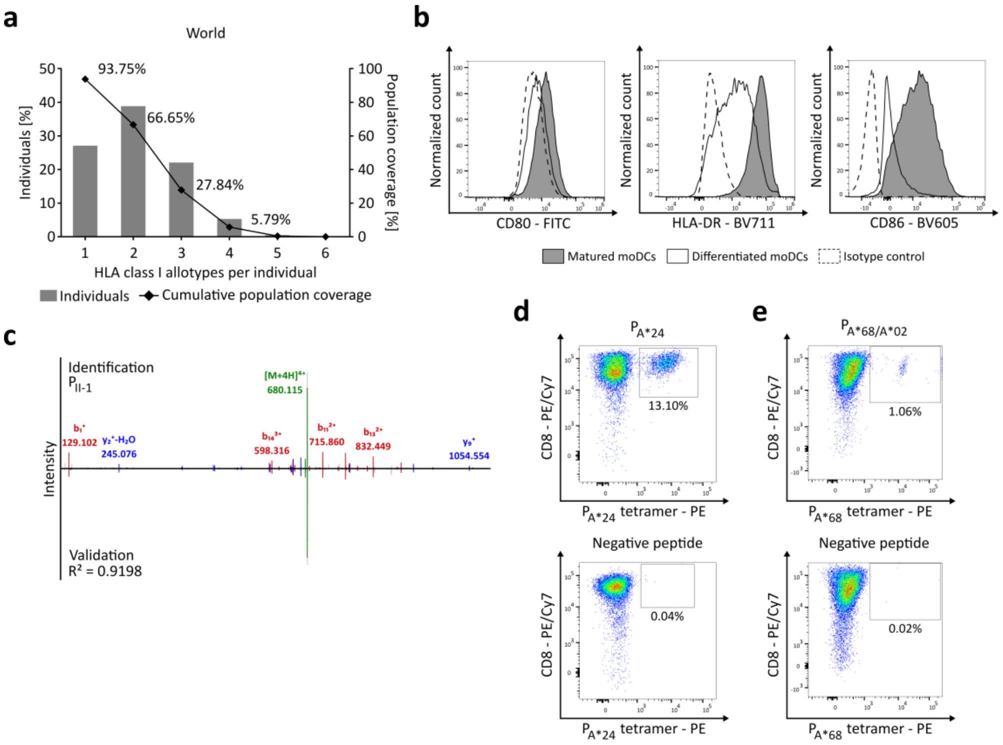

Supplementary Fig. S1: Characterization of DNAJB1-PRKACA-derived T cell epitopes

a, HLA allotype population coverage achieved with predicted HLA class I epitopes within the long P_{II-1} of the DNAJB1-PRKACA protein fusion region compared to the world population. The frequencies of individuals within the world population carrying up to six HLA class I allotypes (x-axis) are indicated as grey bars on the left y-axis. The cumulative percentage of population coverage is depicted as black dots on the right y-axis. **b**, Flow cytometry-based characterization of mature monocyte-derived dendritic cells (moDCs) in comparison to differentiated moDCs and isotype control with indicated surface markers (n=3). **c**, Comparison of fragment spectra (m/z on x-axis) of the experimentally eluted peptide KREIFDRYGEEVKEFLAKAKED (P_{II-1}) extracted from peptide-loaded mature moDCs of healthy volunteer (HV) 6 (identification) with the original synthetic peptide (validation, mirrored on the x-axis), both isotope-labeled on amino acid position 4, 12, and 19. **(d, e)** Representative example of flow cytometry-based characterization of $CD8^+$ T cells of a HV after in vitro artificial antigen presenting cell (aAPC)-priming, shown are **d**, P_{A*24}-specific $CD8^+$ T cells of HV2 primed with HLA-A*24-P_{A*24}-monomer (n=67) and **e**, $P_{A*68/A*02}$-specific $CD8^+$ T cells of HV10 primed with HLA-A*68-$P_{A*68/A*02}$-monomer (n=10). Source data are provided as a Source Data file.

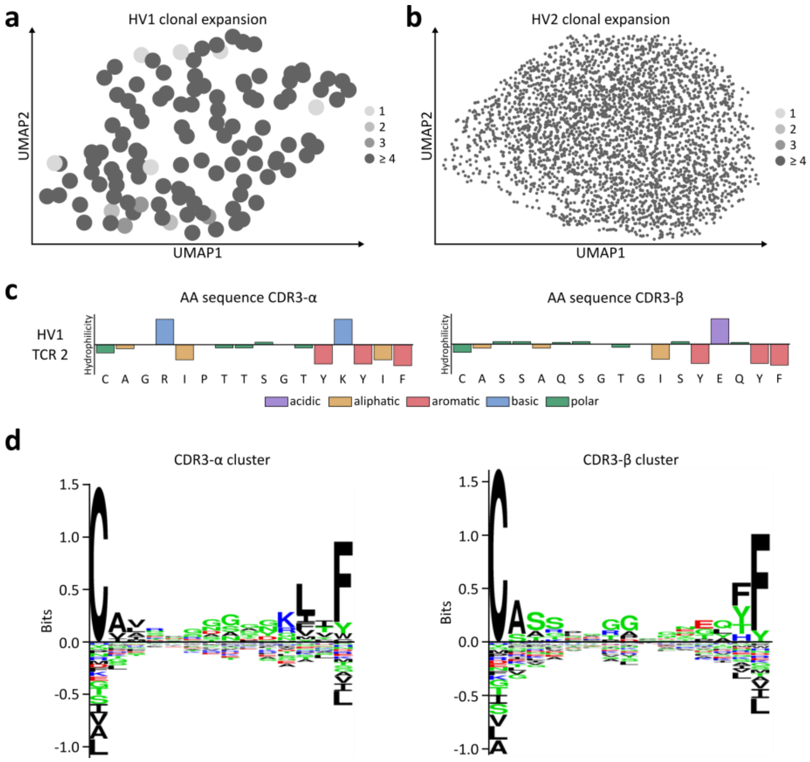

Supplementary Fig. S2: Single-cell TCR sequencing of P_{A*24}-specific CD8$^+$ T cells

(a-c) Single-cell RNA and T cell receptor (TCR) sequencing was performed for tetramer-sorted P_{A*24}-specific CD8$^+$ T cells of two healthy volunteers (HV) after artificial antigen presenting cell (aAPC)-based priming with HLA-A*24-P_{A*24}-monomer. **(a, b)** Uniform Manifold Approximation and Projection (UMAP) plots showing TCR clonality of P_{A*24}-specific CD8$^+$ T cells of **a**, HV1 and **b**, HV2. The color code indicates the number of cells belonging to an expanded clonotype. **c**, Physiochemical properties and amino acid (AA) sequences of the CDR3-α/-β region of the second most frequent TCR clone of HV1. Hydrophilicity according to the Hopp-Woods scale[82] is indicated on the y-axes. AAs are grouped by their physiochemical properties with color code. **d**, Clustering of variable sequences of the CDR3-α and CDR3-β region of a negative dataset containing all described A*24:02 TCRs from the VDJdb database (https://vdjdb.cdr3.net). The clustering was conducted using GibbsCluster 2.0[80]. Source data are provided as a Source Data file.

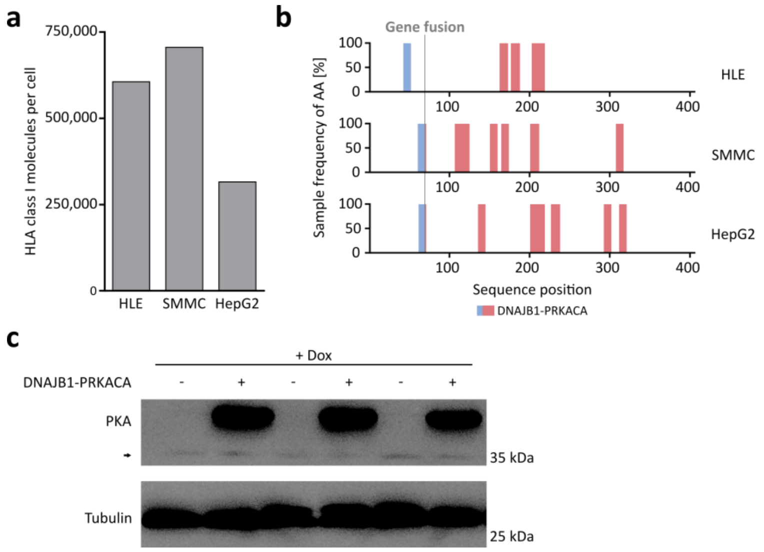

Supplementary Fig. S3: HCC cell lines expressing the DNAJB1-PRKACA fusion protein

a, Flow cytometry-based quantification of HLA class I molecule surface expression for hepatocellular carcinoma (HCC) cell lines HLE, SMMC-7721, and HepG2. **b**, Frequency of amino acids (AA) per sample distributed over the DNAJB1-PRKACA fusion protein sequence of HLA class I ligands identified by mass spectrometry within the HCC cell lines (HLE n=1, SMMC-7721 n=3, and HepG2 n=3) expressing the DNAJB1-PRKACA protein for each cell line separately. **c**, Determination of Doxycycline (Dox)-induced DNAJB1-PRKACA fusion protein expression by immunoblotting of cell lysates of the HCC cell line HLE, used for the loading of monocyte-derived dendritic cells, carrying the DNAJB1-PRKACA Dox-inducible plasmid (+) or the control plasmid (-), after Dox treatment using an anti-Protein Kinase A (PKA) antibody (n=3). The black arrow indicates the endogenous PKA band. Tubulin served as loading control. Source data are provided as a Source Data file.

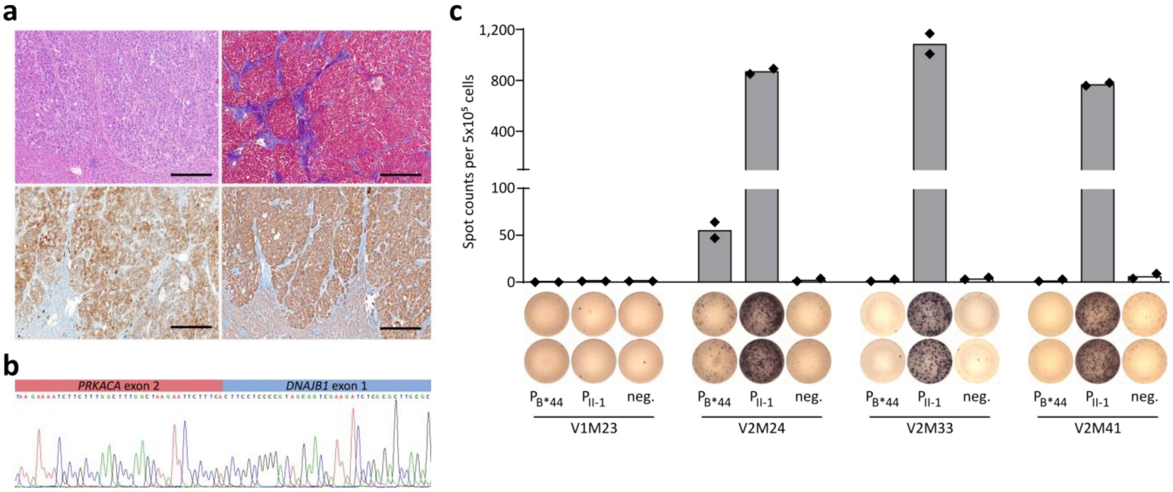

Supplementary Fig. S4: Characterization of primary tumor cells and sequential immunomonitoring of a FL-HCC patient vaccinated with a personalized DNAJB1-PRKACA-derived peptide vaccine

a Representative micrographs were generated within clinical routine diagnostics using accredited and automated antibody/staining panels for the FL-HCC patient at diagnosis. Upper left panel, HE staining (n=1); upper right panel, Masson's Trichrome staining (n=1); lower left panel, CK7 immunohistochemical staining (n=1); and lower right panel, Hepar1 immunohistochemical staining (n=1). Scale bar = 500 µm, magnification x 40. **b**, Sanger sequencing of the reverse transcription polymerase chain reaction (RT-PCR) product for confirmation of the chimera transcript joining the end of exon 1 of DNAJB1 and the start of exon 2 of PRKACA. **c**, IFN-γ ELISPOT assay of peptide-specific T cells from the FL-HCC patient after in vitro stimulation with the vaccine cocktail peptides P_{B*44}, and P_{II-1} in technical replicates (n=2) six weeks after the first vaccination (V1M23), six weeks after the second vaccination (V2M24), 45 weeks after the second vaccination (V2M33), and 84 weeks after the second vaccination (V2M41) compared to the negative peptide (neg.). Source data are provided as a Source Data file.

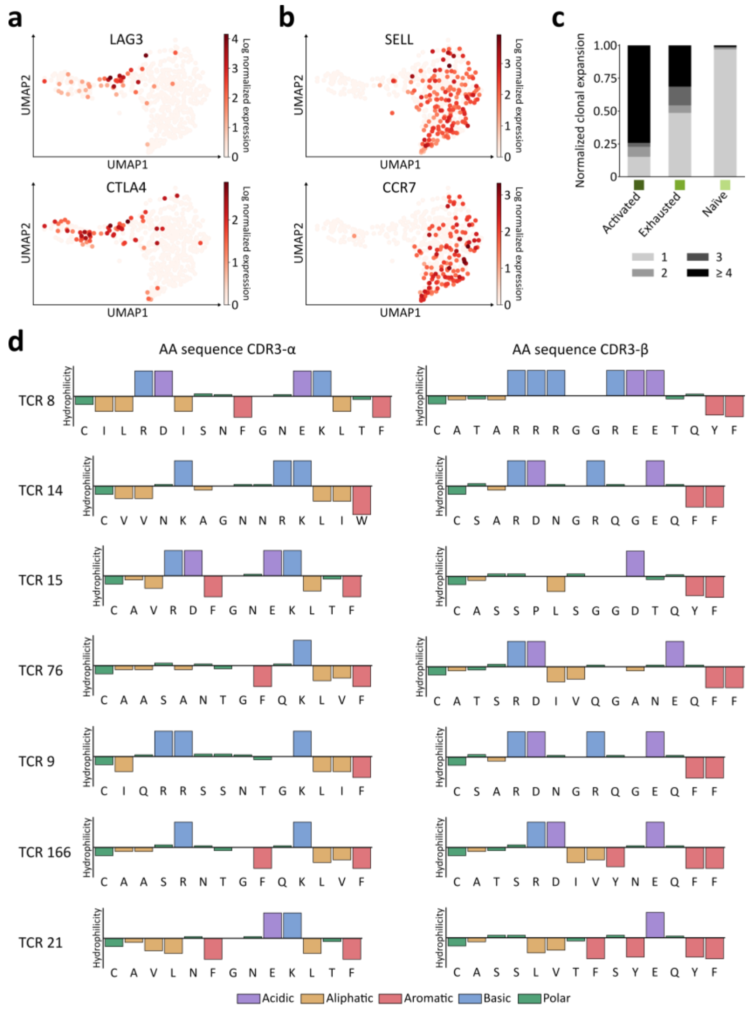

Supplementary Fig. S5: Single-cell RNA sequencing of vaccine-induced P_{II-1}-specific $CD4^+$ T cells

(a-d) Single-cell RNA sequencing analysis of $CD4^+$ T cells sorted from P_{II-1} stimulated PBMCs of the FL-HCC patient 31 weeks after the second vaccination with a personalized DNAJB1-PRKACA-derived peptide vaccine. **a**, Uniform Manifold Approximation and Projection (UMAP) plots depicting log normalized LAG3 and CTLA4 gene expression defining the exhausted/late effector T cell cluster. **b**, UMAP plots depicting log normalized SELL (encoding CD62L) and CCR7 gene expression defining the naïve resting T cell cluster. **c**, Number of cells belonging to an expanded T cell receptor (TCR) clonotype per cell type cluster normalized to cluster size. **d**, Physiochemical properties and amino acid (AA) sequences of the CDR3-α/-β region of TCR clones. Hydrophilicity according to the Hopp-Woods scale[82] is indicated on the y-axes. AAs are grouped by their physiochemical properties with color code. Source data are provided as a Source Data file.

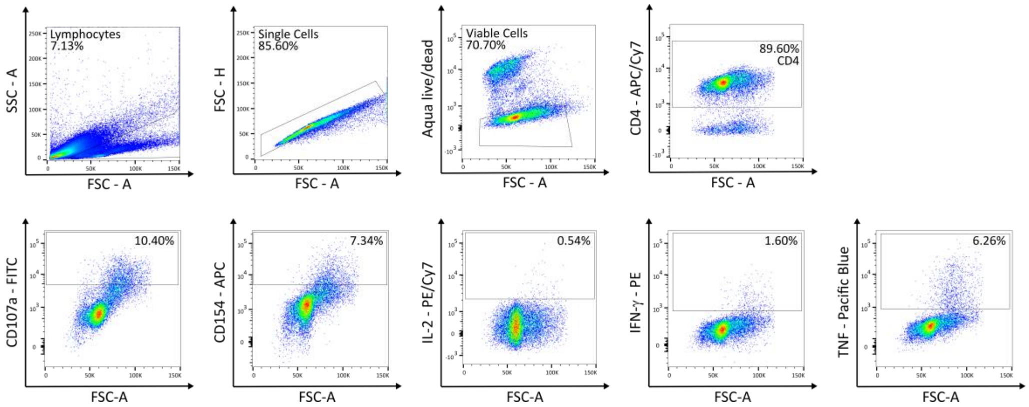

Supplementary Fig. S6: Gating strategy for the ICS evaluation of moDC-based CD4$^+$ T cell priming.

Exemplary sample showing the gating strategy for evaluation of intracellular cytokine stainings (ICS) of monocyte-derived dendritic cells (moDC)-based CD4$^+$ T cell priming. The first gate identifies the lymphocytes (FSC-A vs. SSC-A), which are further gated for single cells (FSC-A vs. FSC-H), viable cells (FSC-A vs. Aqua live/dead), and CD4$^+$ cells (FSC-A vs. CD4-APC/Cy7). CD4$^+$ T cells were analyzed for CD107a (FSC-A vs. CD107a-FITC), CD154 (FSC-A vs. CD154-APC), IL-2 (FSC-A vs. IL-2-PE-Cy7), IFN-γ (FSC-A vs. IFN-γ-PE), and TNF (FSC-A vs. TNF-Pacific Blue) expression and production.

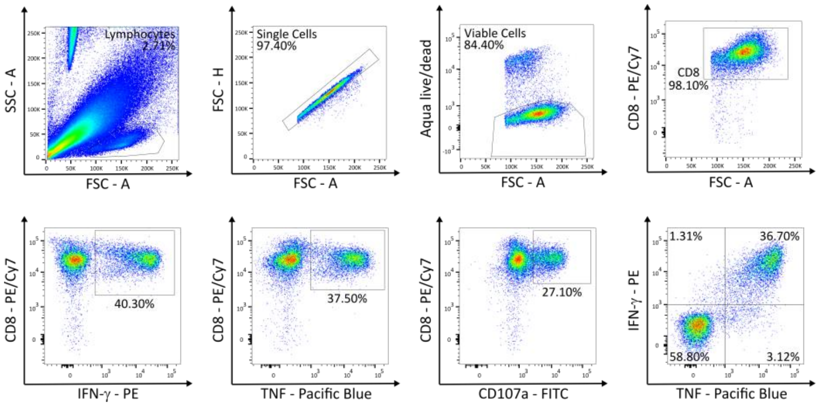

Supplementary Fig. S7: Gating strategy for ICS evaluation of aAPC-based CD8$^+$ T cell priming.

Exemplary sample showing the gating strategy for intracellular cytokine stainings (ICS) of artificial antigen presenting cell (aAPC)-based CD8$^+$ T cell priming. The first gate identifies the lymphocytes (FSC-A vs. SSC-A), which are further gated for single cells (FSC-A vs. FSC-H), viable cells (FSC-A vs. Aqua live/dead), and CD8$^+$ cells (FSC-A vs. CD8-PE/Cy7). CD8$^+$ T cells were analyzed for IFN-γ (IFN-γ-PE vs. CD8-PE/Cy7), TNF (TNF-Pacific Blue vs. CD8-PE/Cy7), CD107a (CD107a-FITC vs. CD8-PE/Cy7), and TNF/ IFN-γ (TNF-Pacific Blue vs. IFN-γ-PE) expression.

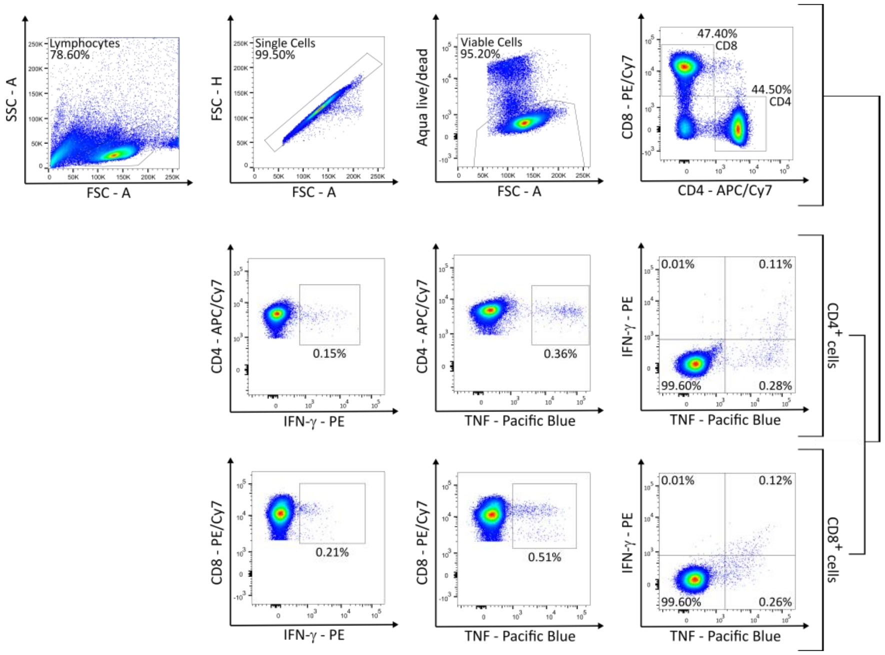

Supplementary Fig. S8: Gating strategy for ICS evaluation of peptide-specific CD4$^+$ and CD8$^+$ T cells from a FL-HCC patient vaccinated with a personalized DNAJB1-PRKACA-derived peptide vaccine.

Exemplary sample showing the gating strategy for intracellular cytokine stainings (ICS) of peptide-specific CD4$^+$ and CD8$^+$ T cells from a FL-HCC patient vaccinated with a personalized DNAJB1-PRKACA-derived peptide vaccine. The first gate identifies the lymphocytes (FSC-A vs. SSC-A), which are further gated for single cells (FSC-A vs. FSC-H), and viable cells (FSC-A vs. Aqua live/dead). The CD4$^+$ and CD8$^+$ T cells (CD4-APC/Cy7 vs. CD8-PE/Cy7) were analyzed separately for cytokine production. CD8$^+$ T cells were analyzed for IFN-γ (IFN-γ-PE vs. CD8-PE/Cy7), TNF (TNF-Pacific Blue vs. CD8-PE/Cy7), and TNF/ IFN-γ (TNF-Pacific Blue vs. IFN-γ-PE) expression, CD4$^+$ T cells for IFN-γ (IFN-γ-PE vs. CD4-APC/Cy7), TNF (TNF-Pacific Blue vs. CD4-APC/Cy7), and TNF/ IFN-γ (TNF-Pacific Blue vs. IFN-γ-PE) expression.

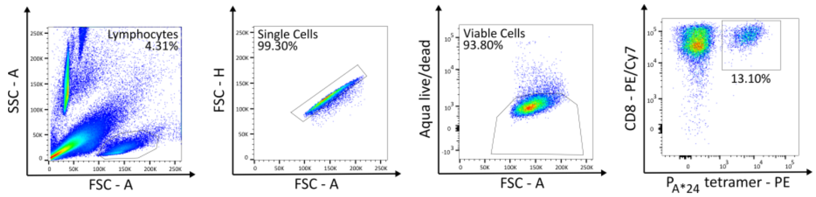

Supplementary Fig. S9: Gating strategy for evaluation of P_{A*24}-tetramer staining of $CD8^+$ T cells.

Exemplary sample showing the gating strategy for evaluation of P_{A*24}-tetramer staining of $CD8^+$ T cells after antigen presenting cell (aAPC)-based priming. The first gate identifies the lymphocytes (FSC-A vs. SSC-A), which are further gated for single cells (FSC-A vs. FSC-H), viable cells (FSC-A vs. Aqua live/dead), and analyzed for $CD8^+$/P_{A*24}-tetramer-specific cells (P_{A*24} tetramer-PE vs. CD8-PE/Cy7).

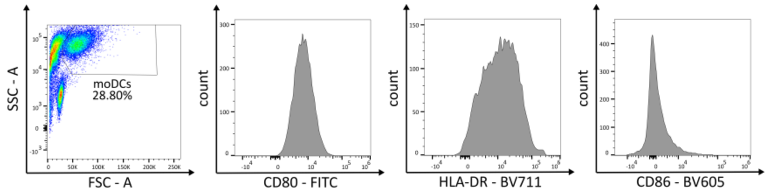

Supplementary Fig. S10: Gating strategy for evaluation of maturated moDC.

Exemplary sample showing the gating strategy for evaluation of maturated monocyte-derived dendritic cells (moDC). The first gate identifies the cells (FSC-A vs. SSC-A), which are further analyzed for CD80 (CD80-FITC vs. count), HLA-DR (HLA-DR-BV711 vs. count), and CD86 (CD86-BV605 vs. count) surface expression.

Supplementary Table 1: HLA typing of patient and healthy volunteers.

Donor[a]	HLA class I[b]	HLA class II[c]
FL-HCC01	A*02:01, B*40:01, *44:02, C*03:04, *05:01	DRB1*04:01, *04:04, DQA1*03:01, DQB1*03:01, *03:02, DPA1*01:03, DPB1*04:01, *06:01
HV1	A*02:01, *24:02, B*13:02, C*06:02	DRB1*07:01, DQA1 *01:03, *02:01, DQB1*02:02, DPA1*01:03, *02:01, DPB1*02:01, *17:01
HV2	A*03:01, *24:02, B*18:01, *52:01, C*07:01, *12:16	n/a
HV3	A*01:01, *02:01, B*44:02, *57:01, C*05:01, *06:02	DRB1*11:04, *13:02, DQA1*01:02, *05:05, DQB1*03:01, *06:09, DPA1*01:03, DPB1*02:01, *04:01
HV4	A*23:18, *31:01, B*14:02, *27:05, C*08:02, *01:02	DRB1*01:02, *11:03, DQA1*01:01, *05:05, DQB1*05:01, *03:01, DPA1*01:03, DPB1*04:01, *13:01
HV5	A*24:02, *31:01, B*07:06, *15:01, C*15:05, *03:03	DRB1*04:05, *13:01, DQA1*01:03, *03:03, DQB1*03:02, *06:03, DPA1*01:03, DPB1*03:01, *04:02
HV6	A*02:01, *24:02, B*07:02, *27:05, C*02:02, *07:02	DRB1*04:01, *13:03, DQA1*03:03, *05:05, DQB1*03:01, *03:02, DPA1*01:03, *02:01, DPB1*04:02, *05:01
HV7	A*02, A*24, B*07, B*27	n/a
HV8	A*24, A*30, B*07, B*15	n/a
HV9	A*02:01, *24:02, B*07:02, *07:05, C*07:02, *15:05	n/a
HV10	A*23:01, *68:02, B*08:01, *44:03, C*07:01, *04:01	DRB1*07:01, *15:01, DQB1*02:02, *06:02, DPB1*04:01

HV, healthy volunteers; n/a, not applicable.

[a] Name of donor.

[b] HLA class I typing.

[c] HLA class II typing.

Supplementary Table 2: Amino acid sequences of TCR CDR3-α/-β and V-, D-, J-genes of P_{A*24}-specific $CD8^+$ T cells.

Donor[a]	Clone[b]	TRAV[c]	AA sequence CDR3-α[d]	TRAJ[f]
HV1	P3-1	1-2	CAAQRRSDKLIF	34
HV1	P3-2	35	CAGRIPTTSGTYKYIF	40
HV2	P4-1	19	CALRQYPAAGNKLTF	17

Donor[a]	Clone[b]	TRBV[c]	AA sequence CDR3-β[d]	TRBD[e]	TRBJ[f]
HV1	P3-1	7-7	CASSLDLSEKLFF	None	1-4
HV1	P3-2	4-1	CASSAQSGTGISYEQYF	1	2-7
HV2	P4-1	7-6	CASSLDLQETQYF	None	2-5

TCR, T cell receptor; AA, amino acid; HV, healthy volunteers.

[a] Name of donor.

[b] Abbreviated T cell clone name.

[c] TRAV or TRBV gene of the TCR.

[d] AA sequence of the CDR3-α or CDR3-β region of the TCR.

[e] TRBD gene of the TCR.

[f] TRAJ or TRBJ gene of the TCR.

Supplementary Table 3: HLA typing of HCC cell lines.

Cell line[a]	HLA class I[b]	HLA class II[c]
HepG2	A*02:01, *24:02, B*35:14, *51:08, C*04:01, *16:02	DRB1*13:02, *16:02, DQA1*01:02, *05:05, DQB1*03:01, *06:04, DPA1*01:03, DPB1*02:01, *04:02
HLE	A*02:06, *24:02, B*15:01, *15:11, C*01:02, *03:03	DRB1*04:05, *12:01, DQA1*03:03, *05:06, DQB1*03:01, *04:01, DPA1*02:02, *02:07, DPB1*02:02, *19:01
SMMC-7721	A*68:02, B*15:03, C*12:03	DRB1*01:02, DQA1*01:01, DQB1*05:01, DPA1*02:01, DPB1*01:01

HCC, hepatocellular carcinoma.

[a] Name of cell line.

[b] HLA class I typing.

[c] HLA class II typing.

Supplementary Table 4: DNAJB1-PRKACA-derived peptide vaccine cocktail.

Sequence[a]	Protein[b]	HLA allele[c]
EIFDRYGEEV	DNAJB1-PRKACA	A*02:01
EEVKEFLAKA	DNAJB1-PRKACA	B*44:02
IFDRYGEEV	DNAJB1-PRKACA	C*05:01
KREIFDRYGEEVKEFLAKAKED	DNAJB1-PRKACA	DPA1*01:03-DPB1*06:01
Pam_3Cys-GDPKHPKSF	XS15	Adjuvant

[a] Amino acid sequence of vaccine peptides.

[b] Source protein.

[c] Predicted HLA allele.

Supplementary Table 5: Amino acid sequences of TCR CDR3-α/-β and V-, D-, J-genes of CD4⁺ T cells of FL-HCC01.

Donor[a]	Clone[b]	TRAV[c]	AA sequence CDR3-α[d]	TRAJ[f]
FL-HCC01	6	27	CAGDRTGGGADGLTF	45
FL-HCC01	8	26-2	CILRDISNFGNEKLTF	48
FL-HCC01	32	29/DV5	CAASRKTGANNLFF	36
FL-HCC01	51	13-2	CAERFSGSEKLVF	57
FL-HCC01	14	12-1	CVVNKAGNNRKLIW	38
FL-HCC01	15	41	CAVRDFGNEKLTF	48
FL-HCC01	76	29/DV5	CAASANTGFQKLVF	8
FL-HCC01	9	26-1	CIQRRSSNTGKLIF	37
FL-HCC01	166	29/DV5	CAASRNTGFQKLVF	8
FL-HCC01	21	36/DV7	CAVLNFGNEKLTF	48

Donor[a]	Clone[b]	TRBV[c]	AA sequence CDR3-β[d]	TRBD[e]	TRBJ[f]
FL-HCC01	6	7-9	CASTLALGANVLTF	None	2-6
FL-HCC01	8	15	CATARRRGGREETQYF	None	2-5
FL-HCC01	32	14	CASSFGIVRTEAFF	None	1-1
FL-HCC01	51	18	CASSPLSGSANEQYF	None	2-7
FL-HCC01	14	20-1	CSARDNGRQGEQFF	None	2-1
FL-HCC01	15	18	CASSPLSGGDTQYF	None	2-3
FL-HCC01	76	15	CATSRDIVQGANEQFF	1	2-1
FL-HCC01	9	20-1	CSARDNGRQGEQFF	None	2-1
FL-HCC01	166	15	CATSRDIVYNEQFF	None	2-1
FL-HCC01	21	7-2	CASSLVTFSYEQYF	None	2-7

Single-cell RNA sequencing analysis of CD4⁺ T cells sorted from P_{II-1}-stimulated PBMCs of FL-HCC01 31 weeks after the second vaccination with a personalized DNAJB1-PRKACA-derived peptide vaccine. TCR, T cell receptor; AA, amino acid.

[a] Name of donor.

[b] Abbreviated T cell clone name.

[c] TRAV or TRBV gene of the TCR.

[d] AA sequence of the CDR3-α or CDR3-β region of the TCR.

[e] TRBD gene of the TCR.

[f] TRAJ or TRBJ gene of the TCR.
