# Supplementary material for: The oncogenic fusion protein DNAJB1-PRKACA can be specifically targeted by peptide-based immunotherapy in fibrolamellar hepatocellular carcinoma
Source: Nat Commun. 2022 Oct 27;13:6401. doi: 10.1038/s41467-022-33746-3 (PMC9613889; doi:10.1038/s41467-022-33746-3)
Supplement: Supplementary file 3 — Reporting Summary [file 41467_2022_33746_MOESM3_ESM.pdf]

nature research

Corresponding author(s): Juliane S. Walz

Last updated by author(s): Sep 22, 2022

Reporting Summary

Nature Research wishes to improve the reproducibility of the work that we publish. This form provides structure for consistency and transparency in reporting. For further information on Nature Research policies, see our Editorial Policies and the Editorial Policy Checklist.

Statistics

For all statistical analyses, confirm that the following items are present in the figure legend, table legend, main text, or Methods section.

n/a	Confirmed	
☐	☒	The exact sample size (*n*) for each experimental group/condition, given as a discrete number and unit of measurement
☐	☒	A statement on whether measurements were taken from distinct samples or whether the same sample was measured repeatedly
☐	☒	The statistical test(s) used AND whether they are one- or two-sided<br>*Only common tests should be described solely by name; describe more complex techniques in the Methods section.*
☒	☐	A description of all covariates tested
☒	☐	A description of any assumptions or corrections, such as tests of normality and adjustment for multiple comparisons
☐	☒	A full description of the statistical parameters including central tendency (e.g. means) or other basic estimates (e.g. regression coefficient) AND variation (e.g. standard deviation) or associated estimates of uncertainty (e.g. confidence intervals)
☐	☒	For null hypothesis testing, the test statistic (e.g. *F*, *t*, *r*) with confidence intervals, effect sizes, degrees of freedom and *P* value noted<br>*Give P values as exact values whenever suitable.*
☒	☐	For Bayesian analysis, information on the choice of priors and Markov chain Monte Carlo settings
☒	☐	For hierarchical and complex designs, identification of the appropriate level for tests and full reporting of outcomes
☐	☒	Estimates of effect sizes (e.g. Cohen's *d*, Pearson's *r*), indicating how they were calculated

Our web collection on statistics for biologists contains articles on many of the points above.

Software and code

Policy information about availability of computer code

Data collection	FACS Canto II cytometer, Orbitrap Fusion Lumos mass spectrometer, GenomeLab GeXP Genetic Analysis System, Chromium Controller instrument 10× Genomics, NextSeq 550 Illumina
Data analysis	GraphPad Prism 9.2.0, The Proteome Discoverer 1.4, FlowJo 10.0.8, GenomeLab GeXP software, bcl2fastq 2.20.0.422 (Illumina)

For manuscripts utilizing custom algorithms or software that are central to the research but not yet described in published literature, software must be made available to editors and reviewers. We strongly encourage code deposition in a community repository (e.g. GitHub). See the Nature Research guidelines for submitting code & software for further information.

Data

Policy information about availability of data

All manuscripts must include a data availability statement. This statement should provide the following information, where applicable:

- Accession codes, unique identifiers, or web links for publicly available datasets
- A list of figures that have associated raw data
- A description of any restrictions on data availability

Source data are provided with this paper. The mass spectrometry proteomics data generated in this study have been deposited in the ProteomeXchange Consortium database (http://proteomecentral.proteomexchange.org) via the PRIDE partner repository under dataset identifier PXD029882 (http://proteomecentral.proteomexchange.org/cgi/GetDataset?ID=PXD029882). The single cell RNA sequencing data generated in this study have been deposited in the NCBI's Gene Expression Omnibus database with the dataset identifier GSE210337 (https://www.ncbi.nlm.nih.gov/geo/query/acc.cgi?acc=GSE210337).

Field-specific reporting

Please select the one below that is the best fit for your research. If you are not sure, read the appropriate sections before making your selection.

- [x] Life sciences
- [ ] Behavioural & social sciences
- [ ] Ecological, evolutionary & environmental sciences

For a reference copy of the document with all sections, see nature.com/documents/nr-reporting-summary-flat.pdf

Life sciences study design

All studies must disclose on these points even when the disclosure is negative.

Sample size	In vitro experiments were conducted with at least 3 healthy donors, as donor HLA types and healthy donor availability were selective factors. The personalized DNAJB1-PRKACA-derived peptide vaccination study was conducted with one patient, due to the rare tumor disease type and patient availability.
Data exclusions	No data were excluded from the analysis.
Replication	All mass spectrometric experiments were analyzed in triplicates. Flow-cytometry based analysis were replicated with at least three healthy donors, except for the HLA-A*68:02 allele here due to the rarity of the allele only one healthy donor was analyzed. All FL-HCC patient related experiments were conducted with one patient due to the rarity of the disease. All attempts of replication were successful.
Randomization	The patient study was conducted with one patient, due to the rarity of the tumor disease type. Therefore no randomization was feasible.
Blinding	The patient study was conducted with one patient, due to the rarity of the tumor disease type. Therefore no blinding was feasible.

Reporting for specific materials, systems and methods

We require information from authors about some types of materials, experimental systems and methods used in many studies. Here, indicate whether each material, system or method listed is relevant to your study. If you are not sure if a list item applies to your research, read the appropriate section before selecting a response.

Materials & experimental systems

n/a	Involved in the study	
	x	Antibodies
	x	Eukaryotic cell lines
x		Palaeontology and archaeology
x		Animals and other organisms
	x	Human research participants
	x	Clinical data
x		Dual use research of concern

Methods

n/a	Involved in the study	
x		ChIP-seq
	x	Flow cytometry
x		MRI-based neuroimaging

Antibodies

Antibodies used

APC/Cy7 anti-human CD4, BioLegend, Cat# 300518, RRID:AB_314086
PE/Cy7 anti-human CD8, Beckman Coulter, Cat# 737661, RRID:AB_1575980
Pacific Blue anti-human TNF-alpha, BioLegend, Cat# 502920, RRID:AB_528965
FITC anti-human CD107a, BioLegend, Cat# 328606, RRID:AB_1186036
PE anti-human IFNγ antibody, BioLegend, Cat# 506507, RRID:AB_315440
APC anti-human IL-2, BioLegend, Cat# 500309, RRID: AB_315096
anti-IFNγ antibody, MabTech, Cat# 3420-3-250, RRID:AB_907283
anti-IFNγ biotinylated detection antibody, MabTech, Cat# 3420-6-250, RRID:AB_907273
anti-CK7, Agilent Dako, Cat# M7018, RRID:AB_2134589
anti-Hepar1, Agilent Dako Cat# M7158, RRID:AB_2335689
FITC anti-human CD80, Biolegend, Cat# 305206, RRID:AB_314502
BV711 anti-human HLA-DR, Biolegend, Cat# 307644, RRID:AB_2562913
BV605 anti-human CD86, Biolegend, Cat# 374214, RRID:AB_2734430
anti-PKAα cat, Santa Cruz, Cat# sc-28315, RRID:AB_628136
anti-GAPD, Cell Signaling, Cat# 5174, RRID:AB_10622025
anti-Tubulin, Merck, Cat# 05-829, RRID:AB_310035
The pan-HLA class I-specific mAb W6/32, the pan-HLA class II-specific mAb Tü 39, and the HLA-DR-specific mAb L243 were produced in-house.

Validation

Antibodies were purchased from the above stated companies. Antibodies are well described and published elsewhere. Information

can be sought from the manufactures website under catalogue number. In-house produced antibodies were validated by flow cytometry.

Eukaryotic cell lines

Policy information about cell lines

Cell line source(s)	HEK293T obtained from the DSMZ, HepG2 obtained from the American Type Culture Collection (ATCC), SMMC-7721 obtained from Woodland Pharmaceuticals, HLE (CVCL_1281) obtained from the Japan Collection of Research Bioresources (JCRB) Cell Bank.
Authentication	HLA typing was conducted for authentication of cell lines.
Mycoplasma contamination	All cell lines were tested negative for mycoplasma contamination.
Commonly misidentified lines (See ICLAC register)	SMMC-7721 is listed as misidentified cell line, it was used due to its specific HLA typing.

Human research participants

Policy information about studies involving human research participants

Population characteristics

For PBMCs of healthy blood donors no demographics were available. For the single vaccinated patient reported in the study the following inclusion and exclusion criteria were applied:

Inclusion Criteria:

Documented diagnosis of advanced malignant disease
Advanced malignant disease without any available standard of care treatment option
Low tumor cell burden
Live expectancy > 6 month
Ability to understand and voluntarily sign an informed consent form.
Ability to adhere to the study visit schedule and other protocol requirements
Eastern Cooperative Oncology Group (ECOG) performance status score of ≤ 2.

Exclusion Criteria:

Pregnant or lactating females.
Treatment regimens inducing severe T cell deficiencies
Treatment-related side effect > CTC grade 2 (CTCAE V5.0
Participation in any clinical study or having taken any investigational therapy, which would interfere with the studies primary and secondary end points within 2 weeks prior to vaccination
Pre-existing auto-immune disease except for Hashimoto thyroiditis and mild (not requiring immunosuppressive treatment) psoriasis

In the study we report on a 13 year old female patient with histologically confirmed FL-HCC. At the time of diagnosis the patient showed no concomitant disease. After first diagnosis (FD) the patient was treated with four cycles of chemotherapy analogous to the PHITT study (PHITT Group F) interrupted by early liver transplant one month after FD, as the tumor was assessed not resectable. Everolimus was used for post transplantation immunosuppression. The patient experienced four relapses after LTx at month 11, 15, 19, and 21 post FD. Tumor manifestations of the first, second and fourth relapse were surgically resected, for the third relapse radiotherapy was applied. Starting at month 16 post FD the patient was treated with Olaparib (poly (ADP-ribose) polymerase (PARP) inhibitor). At month 21 and 23 the patient received two vaccinations of a personalized DNAJB1-PRKACA-derived peptide vaccine.

Recruitment	Healthy blood donors were obtained from the blood bank after informed consent. The personalized DNAJB1-PRKACA-derived peptide vaccination study was conducted with one patient, due to the rare tumor disease type and patient availability, therefore no relevant selection bias ocurred.
Ethics oversight	The study was performed according to the guidelines of the ethics committee at the medical faculty of the Eberhard-Karls-University and at the University Hospital Tübingen (713/2018B02, 406/2019BO2).

Note that full information on the approval of the study protocol must also be provided in the manuscript.

Clinical data

Policy information about clinical studies

All manuscripts should comply with the ICMJEguidelines for publication of clinical research and a completedCONSORT checklist must be included with all submissions.

Clinical trial registration	Personalized vaccination was performed under the protocol NCT05014607. The full trial protocol can be accessed at https://clinicaltrials.gov/ct2/show/NCT05014607.

Study protocol	The single patient vaccinated in this trial was treated under the project (NCT05014607) which aimed to provide personalized multi-peptide vaccination in combination with the TLR1/2 ligand XS15 to individual patients with advanced solid and hematological malignancies without any approved treatment options. Patient treatment was conducted according to §13 Absatz 2b, German-Drug-Law (AMG).
Data collection	Data were collected at day 1 (vaccination, baseline), day 42, day 81, day 140, day 258, and day 358 at the University Hospital Tübingen.
Outcomes	The single patient was monitored for T cell immune responses to vaccine peptides, moreover safety and tolerability of personalized peptide vaccine as well as clinical outcome (imaging and clinical examination) was assessed.

Flow Cytometry

Plots

Confirm that:

- [x] The axis labels state the marker and fluorochrome used (e.g. CD4-FITC).
- [x] The axis scales are clearly visible. Include numbers along axes only for bottom left plot of group (a 'group' is an analysis of identical markers).
- [x] All plots are contour plots with outliers or pseudocolor plots.
- [x] A numerical value for number of cells or percentage (with statistics) is provided.

Methodology

Sample preparation	Sample preparation of the individual flow cytometry experiments are described in detail in the methods part of the manuscript.
Instrument	FACS Canto II cytometer (BD)
Software	FlowJo 10.0.8 (BD)
Cell population abundance	Patient cells have not been enriched or sorted prior to in vitro stimulation. Culturing of peripheral blood mononuclear cells for 12 days in the presence of IL-2 results in T cell enrichment.
Gating strategy	All gating strategies are provided in the manuscript supplement Figure S1-S5.

- [x] Tick this box to confirm that a figure exemplifying the gating strategy is provided in the Supplementary Information.
